# EUS-guided liver biopsy using a modified wet heparin suction technique

**DOI:** 10.1016/j.vgie.2022.05.006

**Published:** 2022-08-11

**Authors:** Ali Zakaria, Abdulrahman Diab, Michael Dowd, Pushpak Taunk, Ali Abbas

**Affiliations:** 1 Division of Advanced Endoscopy, Moffitt Cancer Center, Tampa, Florida; 2 Division of Digestive Disease and Nutrition, Tampa General Hospital, University of South Florida, Tampa, Florida; 3 Division of Digestive Disease and Nutrition, Tampa General Hospital, University of South Florida, Tampa, Florida

**Keywords:** EUS-LB, EUS-guided liver biopsy, EUS-MLB, EUS-modified one-pass, one-actuation wet suction technique, FNB, fine-needle biopsy

## Abstract

Video 1 Description of the technique of EUS-guided liver biopsy using a modified wet heparin suction technique.

Description of the technique of EUS-guided liver biopsy using a modified wet heparin suction technique.

## Background

The field of endohepatology has been evolving recently with multiple studies describing the success and safety of EUS-guided liver biopsy (EUS-LB) in obtaining liver parenchymal tissue. Although the supporting evidence embracing the use of this technique is convincing, variabilities in techniques are still an ongoing field for research.^1^ There is no consensus on the type of needle, depth of needle insertion, number of passes and actuations, or suction technique.

## Technique

Before the procedure, prepare the prerequisite supplies to ensure the procedure is performed in a timely fashion. First, prepare a 19-gauge fine-needle biopsy (FNB) needle (in our center, we used a Franseen-tip needle) by removing the stylet and flushing the needle with heparin flush lock solution without air flush until a few drops are seen at the tip of the needle. Then, prepare a suction syringe loaded to the 20-mL position with vacuum and set aside.

During the procedure, leave the heparin syringe used to flush the needle attached to the needle until just prior to tissue acquisition to preserve the column of heparin within the needle. Insert the FNB needle through the echoendoscope’s working channel, then advance the echoendoscope to the gastric fundus and aim toward the left hepatic lobe. Carefully interrogate the target tissue acquisition site with the endosonographic view using Doppler ultrasound imaging to avoid large blood vessels and bile ducts. Confirm a safe depth of needle insertion of at least 4 cm, and then adjust the needle lock to 4 cm. Detach the heparin syringe from the needle just prior to tissue puncture. Perform one-pass, one-actuation of the left hepatic lobe by introducing the needle through the gastric wall with a quick stroke. Attach the activated empty suction syringe to the proximal port of the needle. Apply suction briefly by rotating the stopcock 180 degrees and notice a heparin splash in the suction syringe, which indicates tissue entrance into the needle by displacing the heparin in the needle retrogradely. Finally, confirm the suction syringe stopcock is closed, and slowly withdraw the needle under Doppler ultrasound imaging guidance to ensure lack of flow tract. Remove the needle from the echoendoscope to allow for tissue acquisition.

After the procedure, use a prefilled 10 mL normal saline solution syringe and gently flush the needle to express the specimen into a cup filled with sterile saline solution. Empty the cup with the specimen over an absorbent pad, and gently flush the core with normal saline solution to clear the blood clots. Use a cotton swab and carefully move the specimen into a formalin cup for final histopathologic evaluation.

Repeat all the aforementioned steps to obtain a sample from the right hepatic lobe. However, advance the echoendoscope to the duodenal bulb, introduce the needle through the duodenal wall, and lock the needle at 5 cm as the depth of needle insertion (Video 1, available online at www.giejournal.org).

## Discussion

The use of EUS-LB has been embraced as a new tool to obtain liver parenchymal tissues; however, variable techniques have been described. Six different suction techniques in EUS-LB have been reported: dry suction, slow pull, wet suction, modified wet suction, wet heparin, and dry heparin.^2^, ^3^, ^4^, ^5^ Our technique is close to the wet heparin suction technique and the modified one-pass, one-actuation wet suction technique (EUS-MLB) with some differences.^6^ In comparison with the wet heparin suction technique, we left the heparin syringe attached to the proximal port of the needle until just prior to liver puncture. This was done to maintain the heparin column in the needle. We detached the heparin syringe, performed the liver puncture, and then attached the suction syringe with brief activation until heparin splash was noticed, indicating entry of specimen into the needle. In comparison with the EUS-MLB technique, we used heparin solution to flush the needle, and we left the syringe attached to the proximal end until just prior to liver puncture. We used a 19-gauge FNB Franseen-tip needle rather than a 19-gauge FNB fork-tip needle, as previous studies reported better outcome using the Franseen-tip needle.^7^^,^^8^ Additionally, we performed a fast one-pass, one-actuation with preset depth of needle insertion to 4 cm in the left hepatic lobe and 5 cm in the right hepatic lobe.

The use of wet heparin suction is superior to the dry heparin suction technique because it yields more complete portal tracts, longer length of the longest piece, higher aggregate specimen length, and less tissue fragmentation.^3^ Therefore, in our technique, we maintained the needle heparin at the maximum level possible prior to liver puncture through 2 modifications. First, we kept the heparin syringe attached to the needle until just prior to liver puncture. Second, we did not apply suction prior to liver puncture, thus preventing any retrograde movement of heparin to the suction syringe prior to actuation. The heparin splash observed in our technique could be used by endoscopists as an indicator that tissue has entered the needle and displaced the heparin from the needle retrogradely. This could decrease the adverse effects of EUS-LB, as fewer passes are needed to ensure that tissue has entered the needle.

## Conclusion

Variable techniques have been described to obtain high-quality EUS-guided liver parenchymal core biopsies. The modifications we applied in our technique include the use of a 19-gauge FNB Franseen-tip needle, a fast one-pass, one-actuation with a preset depth of needle insertion in each hepatic lobe, and brief suction after maintaining maximal heparin column level in the needle, all of which can further decrease tissue fragmentation, increase quantity of complete portal tracts, and increase aggregate specimen length. However, owing to the lack of evidence, further prospective studies are needed to evaluate any advantages of this modified wet heparin technique compared with currently described EUS-LB techniques.

## Disclosure

*All authors disclosed no financial relationships.*

## Supplementary data

Video 1 Description of the technique of EUS-guided liver biopsy using a modified wet heparin suction technique.
